# Dietary free sugar and dental caries in children: A systematic review on longitudinal studies

**DOI:** 10.34172/hpp.2021.35

**Published:** 2021-08-18

**Authors:** Zeinab Mahboobi, Afsaneh Pakdaman, Reza Yazdani, Leila Azadbakht, Ali Montazeri

**Affiliations:** ^1^Department of Community Oral Health, School of Dentistry, Tehran University of Medical Sciences, Tehran, Iran; ^2^Research Centre for Caries Prevention, Dental Research Institute, School of Dentistry, Tehran University of Medical Sciences, Tehran, Iran; ^3^Department of Community Nutrition, School of Nutritional Sciences and Dietetics, Tehran University of Medical Sciences, Tehran, Iran; ^4^Health Metrics Research Center, Institute for Health Sciences Research, ACECR, Tehran, Iran

**Keywords:** Dietary Sugars, Dental Caries, Child

## Abstract

**Background:** Dental caries, as a multi-factorial problem, is prevalent among children. The aim of this review was to assess the association between dietary free sugars (DFS) consumption and dental caries in 6- to 12-year-old children in the recent longitudinal e vidence.

**Methods:** In this systematic review, database search was performed in PubMed, Embase, ISI Web of Science and Scopus using the keywords "diet", "dental caries" and "school children".We considered the articles published in English from 2004 to 2019. After duplicate removal,title, abstract data basell text of all included papers were assessed by two independent reviewers. The quality of included papers was assessed using the Newcastle-Ottawa scale.

**Results:** From 2122 papers, ten longitudinal studies were included. In the included studies, the consumption of 100% juice (daily), candy (more than once a week), and soft drink and sweet drinks (at bedtime) were highly associated with caries in children. In few studies, daily consumption of water and dairy products was reported to be protective. However, some studies reported non-significant association between consumption of different sugary items and dental caries. The quality of included studies was moderate. Heterogeneity was observed in the measurement of caries outcome, and data collection tool for diet assessment, and statistical measure, which impeded the meta-analysis of data.

**Conclusion:** The methodology and results in the longitudinal studies on the association of dietary free sugar consumption and dental caries in schoolchildren were heterogeneous, which urge the need for further standard research protocols in this area.

## Introduction

Dental caries is a multi-factorial disease, and several risk factors and risk indicators influence its incidence and progression. The main risk factors for dental caries are reported to be diet, saliva, fluoride exposure and cariogenic bacteria, which are in contribution to the context of social, behavioral and economic factors. Risk indicators such’ as socio-demographic factors, oral health related knowledge, attitude, and behavior play also important roles on the caries process.^1^ According to the ecological plaque hypothesis, demineralization of tooth structure occurs in dental biofilm as a result of ecological disturbances in response to the external factors such as sugar exposure, inadequate salivary flow and inadequate fluoride exposure.^1^ In the context of “Common Risk Factor” approach to control chronic diseases, diet is emphasized as a risk factor for heart disease, obesity, stroke, cancer, diabetes, as well as dental caries.^2^

In 2001, the strong evidence supporting the relationship between sucrose intake and development of dental caries became weaker in the fluoride era.^3^ Based on the evidence, dietary free sugars (DFS), refer to “all monosaccharides and disaccharides added to foods and sugars naturally present in honey, syrup, fruit juices and concentrates”, increase the risk of dental caries.^4^ Accordingly, reducing the level of free sugars intake to less than 5%-10% of the total energy intake is recommended for better health outcomes.^5^ In 2014, the dose-response relationship between dietary sugars and dental caries was further confirmed, suggesting a very low sugars intake as low as 2%–3% of the total energy throughout life, regardless of the community fluoride level.^6^

A recent review study has shown that children tend to have energy-dense, nutrient-poor snacks in the past few decades which have resulted to an increase in the risk of overweight and obesity, diabetes and dental caries.^7^ In recent years, several studies have been reported on the determinants of dental caries in children and adults. In 2014, Moynihan and Kelly^5^ conducted a review on the impact of sugar on dental caries in children and adults. They included the studies published from 1950 to 2011 with cross-sectional, non-randomized interventional, cohort and population-based designs. However, there is a scarcity in the review studies on *longitudinal* evidence assessing the relationships between sugar consumption and dental caries in children. Our aim in this systematic review was to assess the recent* longitudinal* evidence on the association between DFS consumption and dental caries in 6-12 years old children.

## Methods

This systematic review was conducted using the Preferred Reporting Items for Systematic Reviews and Meta-Analyses guideline (PRISMA)^8^ and was registered in the PROSPERO (Registration ID: CRD42020167627). The PICO question of our study defined as: What is the dental outcome (considering incidence and/or progression) of high intake of DFS in comparison with low intake of DFS in schoolchildren?

### Search strategy

Electronic databases including Embase, PubMed, Scopus, and ISI Web of Science were searched for articles published on the association between “diet” and “dental caries” in “school children” between January 1, 2004 and September 22, 2019.

The search strategy is presented in Table 1. Endnote Reference Manager Software version X8 was used for the management of the articles. Removing the duplicate references, two reviewers (AP and ZM) reviewed the title and abstract of the papers and the full text of the included studies, independently. In the case of disagreement between the reviewers, consensus was reached through discussion.

**Table 1:** Search strategy for databases included in the review

**Embase**	**361**	('diet'/exp OR 'cariogenic diet'/exp OR 'carbohydrate diet'/exp OR ‘carbohydrate loading diet'/exp OR 'free sugar*':ti,ab,kw OR 'added sugar*':ti,ab,kw OR 'sucrose'/exp OR 'sugar'/exp OR 'drinking behavior'/exp OR 'sugar intake'/exp OR 'eating habit'/exp) AND ('dental health'/exp OR 'dmf^a^ index'/exp OR 'dental caries'/exp) AND (2004:py OR 2005:py OR 2006:py OR 2007:py OR 2008:py OR 2009:py OR 2010:py OR 2011:py OR 2012:py OR 2013:py OR 2014:py OR 2015:py OR 2016:py OR 2017:py OR 2018:py OR 2019:py) AND ('article'/it OR 'article in press'/it OR 'conference abstract'/it OR 'letter'/it) AND ([preschool]/lim OR [school]/lim) AND [English]/lim
**PubMed**	**357**	Search ((((((((("Diet"[Mesh]) OR "Diet, Cariogenic"[Mesh]) OR "Diet, Carbohydrate Loading"[Mesh])) OR (("Dietary Sugars"[Mesh]) OR "Dietary Sucrose"[Mesh])) OR (("added sugar"[Title/Abstract]) OR "free sugar"[Title/Abstract])) OR drinking behavior[Title/Abstract]) OR eating habit[Title/Abstract])) **AND** ((((((("Dental Caries"[Mesh]) OR "Tooth Demineralization"[Mesh])) OR "DMF Index"[Mesh]) OR "Oral Health"[Mesh]) OR Caries[Title/Abstract]) OR "Tooth decay"[Title/Abstract]) **Filters:** Congress; Journal Article; Letter; Publication date from 2004/01/01; English; Child: 6-12 years
**Scopus**	**269**	((TITLE-ABS-KEY(diet) OR TITLE-ABS-KEY("cariogenic diet") OR TITLE-ABS-KEY("carbohydrate diet") OR TITLE-ABS-KEY("sugar*") OR TITLE-ABS-KEY(sucrose) OR TITLE-ABS-KEY("eating habit") OR TITLE-ABS-KEY("drinking behavior") OR TITLE-ABS-KEY("added sugar") OR TITLE-ABS-KEY("free sugar"))) **AND** ((TITLE-ABS-KEY("dental caries") OR TITLE-ABS-KEY("tooth demineralization") OR TITLE-ABS-KEY("tooth decay") OR TITLE-ABS-KEY("DMF index") OR TITLE-ABS-KEY(caries) OR TITLE-ABS-KEY("oral health") OR TITLE-ABS-KEY("dental health"))) **AND** ( LIMIT-TO ( PUBYEAR,2019) OR LIMIT-TO (PUBYEAR,2018) OR LIMIT-TO (PUBYEAR,2017) OR LIMIT-TO (PUBYEAR,2016) OR LIMIT-TO (PUBYEAR,2015) OR LIMIT-TO (PUBYEAR,2014) OR LIMIT-TO (PUBYEAR,2013) OR LIMIT-TO (PUBYEAR,2012) OR LIMIT-TO (PUBYEAR,2011) OR LIMIT-TO (PUBYEAR,2010) OR LIMIT-TO (PUBYEAR,2009) OR LIMIT-TO (PUBYEAR,2008) OR LIMIT-TO (PUBYEAR,2007) OR LIMIT-TO (PUBYEAR,2006) OR LIMIT-TO (PUBYEAR,2005) OR LIMIT-TO (PUBYEAR,2004) ) **AND** ( LIMIT-TO ( DOCTYPE,"ar" ) OR LIMIT-TO (DOCTYPE,"cp" ) OR LIMIT-TO ( DOCTYPE,"le" ) ) **AND** (LIMIT-TO (EXACTKEYWORD,"Children" ) OR LIMIT-TO (EXACTKEYWORD,"School Child" ) ) **AND** ( LIMIT-TO (LANGUAGE,"English" ) )
**Web of Science**	**1133**	TOPIC: (diet) OR TOPIC: ("cariogenic diet") OR TOPIC: ("carbohydrate diet") OR TOPIC: ("sugar*") OR TOPIC: (sucrose) OR TOPIC: ("eating habit") OR TOPIC: ("drinking behavior") OR TOPIC: ("free sugar") OR TOPIC: ("added sugar") Indexes=SCI-EXPANDED, SSCI, A&HCI, CPCI-S, CPCI-SSH, ESCI Timespan=All years** AND** TOPIC: ("dental caries") OR TOPIC: ("tooth demineralization") OR TOPIC: ("tooth decay") OR TOPIC: (caries) OR TOPIC: ("DMF*") OR TOPIC: ("oral health") OR TOPIC: ("dental health")

^a^In the Embase database the “dmf” index included both “DMF and dmf” index.

### Eligibility criteria 

The inclusion criteria were (*i*) prospective cohort studies, (*ii*) exposure to each type of sugary items, (*iii*) having tooth decay as an outcome measure, and (*iv*) children aged 6 to 12 years, as study population. The studies published in English in the past 15 years (2004 to 2019) were included. Letters and conference abstracts were included. Publications were excluded if the study subjects had systemic disease and/or disorders. The publications reporting in non-English languages, as well as editorials, reviews, unpublished and grey literature, theses and the papers not fulfilled the inclusion criteria were excluded. The authors of some selected papers were contacted.

### Data extraction

The full texts of all selected papers were reviewed, and the following characteristics for each study were extracted: author names, publication year, sample size, participants’ age at baseline, follow up period, diet assessment tool, caries measurement as outcome, and main results including the reported statistical measures. For each paper, the relevant statistics were reported as hazard ratio (HR), odds ratio (OR), relative risk (RR), incidence rate ratio (IRR), and the mean difference of caries in subjects who had high intake of sugary items compared to those with less intake. The confidence interval was reported for the statistical measure if indicated.

### Risk of bias assessment (quality assessment) 

Two independent reviewers (AP and ZM) assessed the quality of the included papers. The risk of bias was assessed using the Newcastle-Ottawa Scale^9^ for observational studies. The scorings were then checked with the third reviewer (LA), and consensus was reached. The Newcastle-Ottawa Scale contains three domains including the selection of study groups (0-4 points), adequacy of adjustment for confounding (0-2 points), and ascertainment of the outcome of interest (0-3 points). According to the scale guide of the checklist, the maximum score is 9. The papers with the score of 7 and higher were considered to be with *high quality* and otherwise was considered to be with *low quality*.^10^

## Results

### Literature search

**Figure 1:**
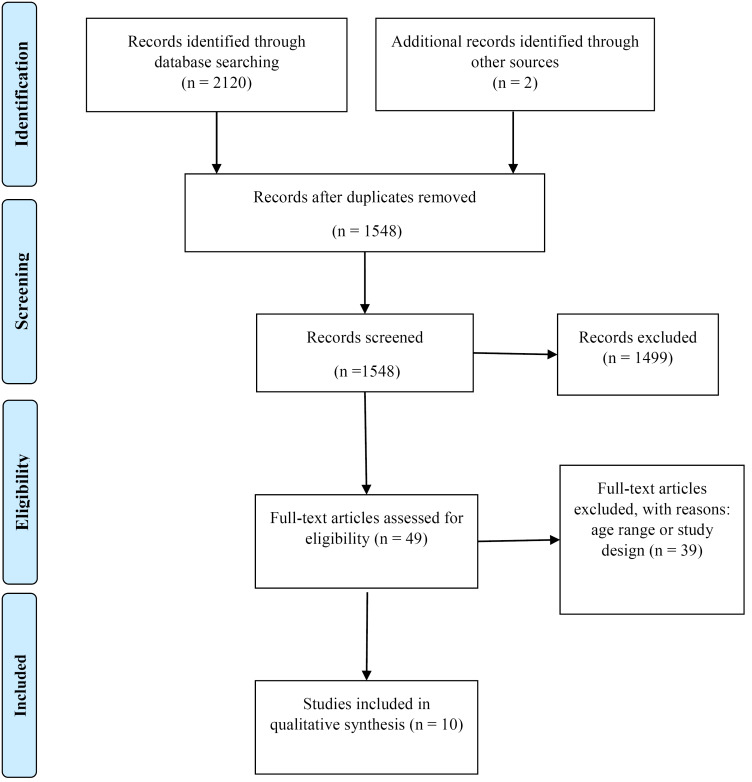
Flowchart of the study according to PRISMA

In total, 2120 publications were identified. Removing 574 duplicates, we assessed the title and abstract of all remaining 1546 papers. Of these, 49 papers were considered for full text review. The studies with cross-sectional design were excluded. After careful review, ten papers were included in the final analysis. The PRISMA diagram is depicted in Figure 1.

### Studies time span

**Figure 2:**
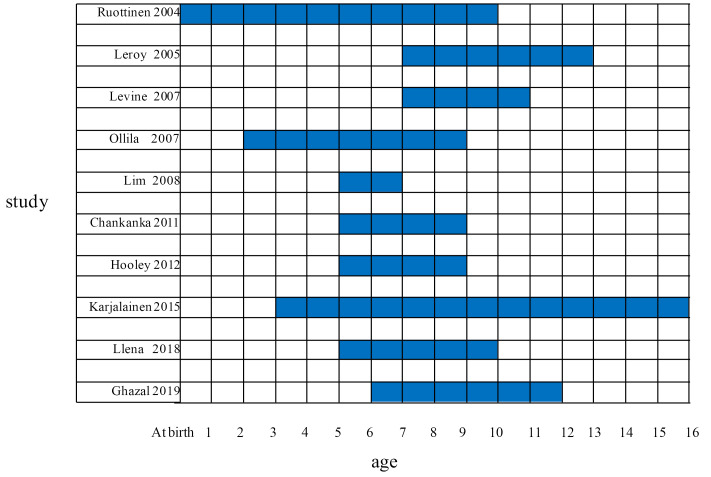
A graphic view of the time span of included studies

The longitudinal studies with different time span were included in the review, and are presented in Figure 2. Due to the nature of longitudinal studies, the studies that covered the age range of children i.e. 6 to 12 years at one end were included. In eight studies, the initiation of study was with 6 years old^11-18^ children and younger, and in two studies, the start was with children over 6 years old.^19,20^ Three studies considered longer time spans; a study started with the children at birth and ended at 10 years of age,^18^ another study started with 3 years old children and last for 13 years,^17^ and one other study started with the children at age 2 and ended at 9 years of age.^11^ The time span of participant children in the included studies is presented in Figure 2.

### Caries measurement

The dental caries as an outcome was reported using different indices:

DMF(Decayed, Missed, and Filled Teeth/Surfaces): Six studies reported DMFT/dmft, including the study of Ollila and Larmas, who reported the dmft as initial vs. manifest caries.^11,13,15,17-19^ Three studies also used the DMFS/dmfs index.^12,16,20^

ICDAS(International Caries Detection and Assessment System): Two papers claimed to use the ICDAS index.^13,16^

Others: In one paper, the experience of caries was reported by parents/guardians.^14^

### Diet assessment

For diet assessment, a variety of scales including diet questionnaires,^11,12,20^ the 24-hour recall questionnaire,^14,19^ the 3-day diet diary,^15,19^ the 4-day food record,^17^ and the Food Frequency Questionnaire (FFQ)^16,18^ were used. One paper^13^ did not report the diet assessment tool. In one study,^19^ two dietary instruments were used to collect dietary data. The details extracted from the selected papers are presented in Table 2.

**Table 2:** Summary of the longitudinal studies reporting the association between diet and dental caries in children

**Author (year)**	**Sample size**	**Age (y)**	**Follow-up (y)**	**Instrument**	**Outcome measure**	**Main results**	**Quality score** ^a^
Ruottinenet al, 2004^18^	66	at birth (infancy)	10	FFQ	dmft+DMFT	Sucrose intake (SI) [high vs. low] and mean ± SDof caries as dmft (1.4 ± 2.0 vs. 0.5 ± 1.1, *P* = 0.01); Sucrose intake (SI) [high vs. low] and mean ± SD of caries as dmft+DMFT (3.9± 3.9 vs. 1.9 ± 2.5, *P* = 0.03)	4
Leroy et al, 2005^20^	2268	7.08 (mean)	6	Structured questionnaire	DMFS^d^	Sweets/biscuits, sweet snacks, sugar-containing drinks, NS.	9
Levineet al, 2007^19^	437	7	4	24-hour recall & 3-day food diary	d_2_mft/D_2_MF^d^(cavitation)	Bedtime consumption of NMES drink at age 7-11 years with D_2_MF in 11-15 years old (<1 vs.1+), *P* = 0.031^b^; Bedtime consumption of NMES drink at age 7-11 years with caries in 11-15 years old, OR: 1.92 (CI not reported), *P* = 0.033^c^; Dairy product consumption at age 11-15, OR: 0.61(CI not reported), *P* = 0.049	6
Ollilaet al, 2007^11^	183	2	7	Questionnaire	Initial caries (Enamel) vs. Manifest caries (Dentine)	Candies more than once a week with caries onset in tooth 55, HR: 6.83 (CI not reported), *P* < 0.001; Candies more than once a week with caries onset in tooth 75, HR: 8.18 (CI not reported), *P* < 0.001; Candies more than once a week with the caries onset in tooth 16, HR: 3.8 (CI not reported), *P* = 0.025 Mean survival times for primary 2^nd^ molars in children with candy consumption more than once a week compared with no use (7.83 vs. 10.06 years), *P* < 0.001; Mean survival times for permanent 1^st^molars in children with candy consumption more than once a week compared with no use (10.03 vs. 10.21 years),NS	7
Limet al, 2008^16^	369	3 - 5	2	FFQ	d_2_mfs (Dentine), ICDAS New filled surface	Soft drink consumption (low at baseline changed to high) with caries increment IRR: 1.75 (1.16, 2.64), *P* < 0.05; Soft drink consumption (low at baseline changed to high) with new filled surface IRR: 2.67 (1.36, 5.23), *P* < 0.05; Soft drink consuming (high at baseline and follow up) with new filled surface IRR: 2.68 (1.44, 4.96, *P* < 0.05) Total sugar intake (middle-high compared with low)^d^and caries, NS	7
Chankankaet al, 2011^15^	198	5	4	3-day food diary	d_2-3_ f/D_2-3_ F (cavitation)	Processed starch at snack time OR: 3.87 (0.93, 16.16), *P* < 0.07; [Milk. 100% juices, juice drinks, powder-sugared beverages, regular (sugared) soda pop, diet soda pop, sports drinks, and water. Sugar-based desserts, candy, added sugar (table sugar, honey, brown sugar, etc), baked starch with sugar (cookies, etc)], NS	7
Hooley et al, 2012^14^	4149	4 – 5	4	24-hour recall	Caries experience (cavity or filling or extraction) (yes/no)	Sweet drink (wave 3) OR: 1.1, *P* = 0.01; Sweet drink (wave 2), NS	7
Karjalainenet al, 2015^17^	148	3	13	4-day food record	d_3_mft/ D_3_MFT (cavitation)	The mean ± SE of caries in high SI-group was higher than low SI-group (*P* = 0.046) High sucrose intake **(**≥10% E**)** at age 3 was associated with higher risk of caries at age 6, 9, 12, 16.	7
Llenaet al,^e^ 2018^13^	206	10	5	NR	DMFT and DMFT (6^st^) (cavitated vs. non-cavitated), ICDAS	No sweet consumption with cavitated & non-cavitated lesions (ref. yes) PR: -0.59 (-1.14, -0.01), *P* = 0.04; No soft drink with cavitated & non-cavitated lesions (ref. yes) PR: -0.58 (-1.20, -0.02), *P* = 0.04; No soft drink with cavitated lesions (ref. yes) PR: -0.69 (-1.43, -0.03), *P* = 0.03; No soft drinks with cavitated lesions (all permanent teeth) (ref. yes) PR: -0.76 (-1.44, -0.07), *P* = 0.03; No soft drinks with cavitated& non-cavitated lesions (all permanent teeth) (ref. yes) PR: -0.58 (-1.17, -0.01), *P* = 0.04 Sweets, pastry, snacks, NS	8
Ghazalet al, 2019^12^	98	5.9	6	Questionnaire	DMFS (6s)	Daily consumption of 100% juice HR: 1.54 (0.99, 2.40), *P* = 0.053; Daily consumption of water HR: 0.44 (0.26, 0.74), *P* = 0.002 Daily consumption of milk, added-sugar beverages, daily frequency of candy and/or gum consumption, consumption of candy and/or gum, NS	7

Abbreviations: HR, hazard ratio; PR, prevalence ratio; OR, odds ratio; IRR, incidence rate ratio; FFQ, Food Frequency Questionnaire; ICDAS, international Caries Detection and Assessment System; NS: not significant. ^a^New Castle Ottawa Scale; ^b^ Mann-Whitney U test;^c^Multivariate analysis;^d^Cariogenic solid food; ^e^ Historical cohort.

### Main findings

Among 10 selected studies, two studies showed non-significant associations between DFS consumption (food/drink) and dental caries development.^15,20^ Several studies showed a mix of significant and non-significant associations between DFS intake and dental caries,^11,13,14,16,17,19^ one indicated a borderline association,^12^ and one showed a significant relationship between sucrose intake and dental caries onset.^18^ The statistics used to report the association between caries outcome and sugar consumption were: (*i*) hazard ratio (HR),^11,12,17^ (*ii*) odds ratio (OR),^14,15,19^ (*iii*) prevalence ratio (PR),^13^ and (*iv*) incidence rate ratio (IRR).^16^ The summary of findings are presented as follow.

### Association between DFS and dental caries in children presented as HR

Ghazal et al conducted a study on 98 African-American school-aged children in a non-fluoridated community. They reported that daily consumption of 100% fruit juice had marginally significant association with a shorter time of caries experience in the permanent dentition, after adjusting for the consumption of milk and sugary beverages (HR=1.54; 95% CI: 0.99-2.40; *P* = 0.053), though its impact was marginally significant.^12^

In a 7-year follow-up study conducted by Ollila and Larmas on Finnish children, the consumption of candies and inadequate oral hygiene at the age of two was reported as risk factors for caries development in both primary and permanent molars. In this study, the children eating candies more than once a week had higher HR on the development of new caries in deciduous molar teeth (tooth 55 [HR = 6.83, *P* < 0.05] and 75 [HR = 8.18, *P* < 0.05]), and permanent teeth (tooth 16) (HR = 3.13, *P* < 0.05).^11^

### Association between sugar consumption and dental caries in children presented as OR

Chankanka et al,^15^ Levine et al,^19^ and Hooley et al^14^ reported OR for the associationbetween sugar consumption and dental caries. In the former study, on a sample of 198 Caucasian children, 37% had new carious lesions, which were significantly associated with cavitated and non-cavitated caries experienced at the age of 5, frequent consumption of processed starch at snack time, being older, and having less frequent tooth-brushing. As a subsidiary finding, consumption of processed starch at snack time had a marginal significant association with dental caries (OR = 3.87; 95% CI: 0.93-16.16, *P* = 0.07). No significant association was reported between having other sugary snacks and dental caries.^15^

In the study conducted in 2007 by Levine et al, 608 children aged 7-11 years were followed up for 4 years. The 7-11 years old children who had sugary beverages before bedtime were 2 times more likely to develop caries later at the age of 11-15 (OR = 1.92, *P* = 0.033). In addition, moderate consumption of dairy products and daily tooth brushing had protective effects against caries development (OR = 0.61, *P* = 0.049).^19^ Hooley et al in 2012 used the longitudinal data on 4-5 years old Australian children, and found that the dental problems reported by caregivers were significantly associated to consumption of sweet drinks (OR = 1.1, *P* = 0.01).^14^

### Association between sugar consumption and dental caries in children presented as PR and IRR

Other studies reported either PR or IRR. In a 5-year historical cohort on 10-year-old children, Llena and Calabuig found a significant relationship between dental caries, as measured by various indices, and diet. According to multivariate analysis on the data of 206 children aged 10 years in 2018, not having cariogenic diets particularly soft drinks were found as significant protective factors against dental caries, as measured by DMFT (all teeth), and the DMFT of the first molars when both cavitated and non-cavitated caries were considered.^13^

Lim et al conducted a longitudinal study on 369 low socio-economic status African-American children aged 3-5 years in Detroit, and reported IRR for the associations. The results of their two-year follow-up showed a significant relationship between the intake of soft drink, as a risk factor, and the development of new cavitated lesions. They found that the children who changed the pattern of soft drink consumption from low, at baseline, to high, after 2 years, experienced a higher incidence rate of dental caries [IRR = 1.75; 95% CI: 1.16-2.64]. For those who changed from low consumption of soft drink to high, the IRR for new filled surfaces was 2.67 (95% CI: 1.36-5.23), and for those who the consumption level was reported as high at baseline, and remained high at the follow up, the IRR for new filled surfaces was 2.68 (95% CI: 1.44-4.96).^16^

Ruottinen et al^18^ and Karjalainen et al^17^ reported mean (SE) for dental caries. In 2004, Ruottinen et al conducted their analysis on the data obtained from the STRIP (Special Turku Coronary Risk Factor Intervention Project) study, within which sixty-six children were followed up for 10 years. Their results indicated that the mean score for caries was significantly higher in children with higher sucrose intake, compared to those with low sucrose intake. Similar findings were reported by Karjalainen et al in 2015.

### Non-significant association between DFS and dental caries

In some studies, insignificant association was reported between having sugary items (including sweet drinks, sweets/candy and added sugar) and dental caries.^12,15,20^ The 6-year follow up study by Leroy et al in 2005 on 7 years old children showed no association between dental caries development and consumption of sweets, biscuits, sweet snacks and sugar-containing drinks.^20^ In the study conducted by Chankanka et al, no association was reported between added sugars intake and dental caries.^15^ In the study performed by Ghazal et al, the positive relationship between 100% fruit juice and the shorter time to caries development was marginally significant (*P* = 0.053), and no significant association was found between the other dietary items with DFS and caries development.^12^ However, in the most of included studies, a mixture of significant and non-significant association was reported between the consumption of foods and drinks with DFS and dental caries.^11,13,14,16,17,19^

### Risk of bias

The risk of bias in the included papers were evaluated according to the Newcastle-Ottawa scale, and is presented as quality score in Table 2. Except two studies,^18,19^ all longitudinal studies were scored as high quality.

## Discussion

In the current review, we reported the longitudinal evidence on the impact of DFS on dental caries in children. The more consumption of sugary foods and drinks, particularly before bedtime, was associated with higher risk of dental caries. However, some studies did not show any significant association between having sweet food and dental caries in children. In some studies, the consumption of processed starch was highly cariogenic. The consumption of water and dairy products showed to be with protective effects against dental caries development in children.

Although our review confirmed significant association between sweet consumption and dental caries, some studies reported no significant association in this regard. It is noteworthy that the quality of studies with non-significant results^15,20^ was scored as “high” according to the scale used. In the studies that reported a significant association, the frequent sweet consumption, especially more than 10% of total energy intake, was associated with caries.^17,18^ This finding was in line with the recent health recommendation to limit the sugar consumption to less than 5% of the total energy intake.^4^ Other longitudinal studies on 6-12 years old children showed that soft drink consumption was associated with high level of caries in deciduous dentition^14,16^ and permanent teeth.^13^ In the longitudinal studies started on the children with 6 years of age and older, having frequent consumption of candy was significantly associated with caries development. Sweet beverage consumption especially before bedtime was associated with higher caries.^19^

This review study confirmed the impact of sugar-sweetened beverages on caries development.^13,14,16-19^ The review study conducted by Bleich and Vercammen on the unhealthy effects of sugary drinks on children’s general and oral health, sweet drinks were found to increase the chance of overweight/obesity and development of dental caries.^21^ However, a recent review by Lueangpiansamut et al,^22^ showed no association between sugar-sweetened beverage consumption, including soft drinks and other sugary drinks, and caries development in the primary and permanent dentition. A large body of evidence suggests that sugar-sweetened beverage consumption should be decreased to promote the children’ dental health.^21^

Moreover, bedtime consumption of sugary foods and drinks is reported to serve as a risk factor for dental caries. There is evidence that sugar consumption before bedtime increases the risk of caries, which is due to the reduced saliva flow and sustained low plaque pH. The study of Levine et al also found similar results, indicating that bedtime consumption of sweet drinks (non-milk extrinsic sugars) at the age of 7-11 years was significantly associated with caries in 11-15 years of age.^19^ This finding was in line with those reported in a recent systematic review performed by Baghlaf et al who investigated the relationship between dental caries experience and consumption of foods and drinks, containing free sugars at bedtime in children aged 3-16 years.^23^ They found a positive association between dental caries and free sugars consumption at bedtime in children. In 2018, Taqi et al also found that the children who consumed cariogenic foods and drinks between the main meals and within two hours before bedtime had significantly higher mean caries, as measured by the DMFT index, compared to the children without such habits.^24^

In one study, marginally significant association was reported between the consumption of processed starch in the snack time and dental caries among 6 years old children and younger.^15^ The effect of starch on dental caries was further emphasized in a recent review study.^25^ These findings was in line with those reported in a narrative review conducted by Hujoel and Lingström in 2017. They found that the fermentable carbohydrates were responsible for caries development. The susceptibility of teeth to dental caries in the presence of fermentable carbohydrates, including sucrose, glucose, fructose, lactose, maltose and starch, has been discussed in the literature.^26^ As starch is not included in the DFS definition, this finding may be considered as subsidiary.

Some of the included studies showed no significant association between having various sugary items and dental caries,^12,15,20^ mainly those conducted among children in minority groups and low socio-economic settings. One justification for such non-significant relationships may be the nature of caries development, which is time consuming and multi-factorial. The impact of diet on dental caries might be influenced by some predisposing factors such as fluoride exposure, oral hygiene practice, and saliva secretion. Moreover, as shown by a review study,^27^ oral health is deteriorated in low-income communities. Considering the multi-factorial nature of dental caries, there is a necessity for diet advice along with other preventive measures to control caries in schoolchildren, which is in line with those reported in other review studies on dental caries progression in children.^28^

Studies included in our review were heterogeneous in terms of indices used to measure the caries outcome (i.e., DMFS, dmfs, DMFT, dmft, and ICDAS/BASCD criteria). In addition, various dietary tools were used such as 24-hour recall, FFQ, 4-day food record, 3-day diary, and diet questionnaire, to assess diet status. There was also heterogeneity between the studies in terms of study subjects and follow up periods. Besides, due to data variability in the type and quantity of sugary items, settings and units of outcome measures, any comparison between the outcome measures was difficult, and thus meta-analysis was not possible.

One of the strengths of our review was the selection of the longitudinal studies to clarify the impact of sugar consumption on dental caries in children. Although several review studies found associations between diet and dental caries, there was a scarcity in the review studies on the longitudinal evidence, especially in recent years. In the present study, the Newcastle-Ottawa scale was used to assess the risk of bias in the included studies.

Moreover, the findings extracted from included studies showed a magnitude of association between having sugary food and/or drink and dental caries. A novelty of our review was the presentation of different statistics used in the longitudinal studies based on risk indicators, such as OR, PR, IRR and HR. In order to facilitate the comparison, it is important to consider that PR is equivalent to risk ratio in cohort studies, when the outcome is common.^29^ Two other indicators, including HR and IRR, report the chance of occurring an event, after adjusting for time in the longitudinal studies.

We acknowledge that due to huge amount of information on this topic, in this study we only reviewed the recent longitudinal evidence. However, our review is valuable especially in the fluoride era to assess the impact of sugar on dental caries in children. Due to the nature of longitudinal studies, we included the studies that both started in early childhood and initiated after this period. Therefore, the results of our review should be interpreted with caution. For future studies, it is suggested to use a standardized set of measurement tools, including diet assessment specific for sugary snack consumption and dental caries.

## Conclusion

Our findings indicated that the daily consumption of 100% juice, candy use more than once a week, and soft drink and sweet drink at bedtime were all associated with higher risk of dental caries. The non-significant association between the consumption of sweet food and dental caries was reported in some of the included studies. Other subsidiary findings indicated that having processed starch increase the risk of caries, while the consumption of water and dairy products have some protective effects. As the type of sugary items was not standardized across the studies, and dental caries were measured applying different indices, the findings our review should be interpreted with caution. Further research is needed to assess the magnitude of association between common sugary items and dental caries using a standardized tool.

## Acknowledgements

The authors appreciate PROSPERO for reviewing and approving our study protocol (Registration number: CRD42020167627) and that the Doi: 10.21203/rs.2.24169/v1 is allocated to the pre-print through Research Square website.

The authors acknowledge the help of Dr. Akbar Shafiee Specialist in Community Medicine (Department of Cardiovascular Research, Tehran Heart Center, Tehran University of Medical Sciences, Tehran, Iran) in guiding the database search of this study.

## Funding

There was no grant related to this study and was done as part of PhD thesis supported by Tehran University of Medical Sciences, Tehran, Iran.

## Competing interests

The authors declare that they have no competing interests.

## Ethical approval

As all data used in this systematic review have already been published, additional approval from ethical committee was not applicable.

## Authors’ contributions

AP contributed to the conceptualization and study design, data collection and interpretation, manuscript drafting and its editing. RY’s major role was conceptualization and study design, help in interpretation of the data and drafting. ZM helped greatly in data extraction, interpretation, manuscript writing and revisions. LA helped in data extraction, quality assessment of papers, interpretation of data and preparation of the final draft, AM helped in data extraction, manuscript revision, and interpretation of data. All authors have read and approved the submitted and revised final version of the manuscript and confirm that it is not published elsewhere and is not copied from other papers.

## Disclaimer

The authors claim that this manuscript has not been published in any journal and no part of this paper is copied from other sources.

## Availability of data and material

The data is available from corresponding author (AP) upon receiving the request.
